# Human Genome-Wide RNAi Screen for Host Factors That Modulate Intracellular *Salmonella* Growth

**DOI:** 10.1371/journal.pone.0038097

**Published:** 2012-06-11

**Authors:** Joshua M. Thornbrough, Tom Hundley, Raphael Valdivia, Micah J. Worley

**Affiliations:** 1 Department of Biology, University of Louisville, Louisville, Kentucky, United States of America; 2 Department of Microbiology and Immunology, University of Louisville, Louisville, Kentucky, United States of America; 3 Department of Molecular Genetics and Microbiology, Duke University, Durham, North Carolina, United States of America; Indian Institute of Science, India

## Abstract

*Salmonella enterica* is a bacterial pathogen of humans that can proliferate within epithelial cells as well as professional phagocytes of the immune system. While much has been learned about the microbial genes that influence the infectious process through decades of intensive research, relatively little is known about the host factors that affect infection. We performed a genome-wide siRNA screen to identify host genes that *Salmonella enterica* serovar Typhimurium (*S. typhimurium*) utilizes to facilitate growth within human epithelial cells. In this screen, with siRNAs targeting every predicted gene in the human genome, we identified 252 new human-host-susceptibility factors (HSFs) for *S. typhimurium*. We also identified 39 genes whose silencing results in increased intracellular growth of *S. typhimurium.* The HSFs identified are regulated most centrally by NFκB and associate with each other through an extremely dense network of interactions that center around a group of kinases. Most genes identified were not previously appreciated as playing roles in the intracellular lifecycle of *S. enterica*. Numerous HSFs identified with interesting characteristics that could play plausible roles in mediating intracellular microbial growth are discussed. Importantly, this study reveals significant overlap between the host network that supports *S. typhimurium* growth within human epithelial cells and the one that promotes the growth of *Mycobacterium tuberculosis* within human macrophages. In addition to providing much new information about the molecular mechanisms underlying *S. enterica*-host cell interplay, all 252 HSFs identified are candidates for new anti-microbial targets for controlling *S. enterica* infections, and some may provide broad-spectrum anti-microbial activity.

## Introduction

*S. enterica* is a Gram negative, enteric bacterial pathogen that can infect diverse hosts including birds, reptiles and mammals. *S. typhimurium* causes a self-limiting gastroenteritis in humans whereas the closely related *S. enterica* serovar Typhi (*S*. *typhi*) causes typhoid fever, a frequently fatal systemic disease. *S. enterica* infection is a major public health problem causing more than one billion new human infections each year that lead to more than three million deaths [1]. The problem is greatly exacerbated by the emergence of multi-drug resistant strains [2]. In addition to public health concerns, *S. typhimurium* is also studied because it is a model pathogen without parallel for dissecting basic pathogenic processes.

*S. enterica* promotes its virulence with a class of widespread secretion systems termed type III (TTSS). These sophisticated molecular devices function as ‘molecular syringes’ that span the bacterial envelope and inject proteins into host cell cytosol to subvert various cellular functions. *S. enterica* possesses two distinct TTSSs encoded by *Salmonella* pathogenicity islands 1 and 2 (SPI-1 and SPI-2). *S. enterica* primarily utilizes SPI-1 to invade cells and invoke the inflammatory response [3]–[5], and subsequently SPI-2 to proliferate within cells [6]–[8] and to manipulate the migratory properties of phagocytes [9], [10].

Most studies relating to *S. enterica*-host cell interactions to date have focused on microbial virulence factors. While much has been learned about microbial factors that promote growth within hosts, very little is known about the host cell factors that *S. enterica* uses to facilitate its proliferation. The identification of these HSFs could shed much light on the molecular mechanisms underlying the ability of *S. enterica* to cause disease and could also serve as a large class of novel, anti-microbial targets.

New antibiotics are desperately needed to control infectious diseases caused by *S. enterica* as well as by related intracellular pathogens. Multi-drug resistant strains of *S. typhi* are now commonplace [1]. Since 1989, strains of *S. typhi* resistant to chloramphenicol, ampicillin, and trimethoprim have caused numerous outbreaks [11]. As a result of the widespread dissemination of such strains, chloramphenicol was withdrawn as the first-line drug for typhoid fever and replaced with fluoroquinolones and third generation cephalosporins [12]. However, outbreaks of typhoid fever caused by strains resistant to nalidixic acid and ciprofloxacin have become endemic in the Indian subcontinent and have also been reported in the US and UK among other developed countries, reflecting the emergence of a global problem [13]. The presence of a plasmid-borne integron in ciprofloxacin-resistant *S. typhi* may soon produce widespread instances of nearly intractable typhoid fever [13]. The gravity of this public health problem is heightened by the fact that there are few druggable *S. enterica* targets remaining to inhibit. A recent systematic network analysis of *S. typhimurium* metabolism in the murine model of typhoid fever determined that nearly all *S. typhimurium* enzymes are non-essential, due to extensive metabolic redundancies [14]. Of those that are essential, nearly all belong to metabolic pathways already inhibited by current antibiotics [14]. There is thus a pressing need to develop a new class of drug targets which *S. enterica* and related pathogens will be unable to quickly evolve ways to overcome.

Targeting HSFs might prove useful in treating *S. enterica* infections because *S. enterica* is primarily intracellular in the systemic phase of disease, as gentamicin does not resolve infections, and mutants sensitive to macrophage killing are avirulent [15]. Second, point mutations, which are the most frequent form of mutation, exemplified by ciprofloxacin resistance being produced by point mutations in *gyrA* and *parC* [13], would not likely enable the bacteria to overcome this new class of drug, as resistance would likely require multiple, large genetic changes. Unfortunately, only a few HSFs for *S. enterica*, most notably AKT1, have been reported [16].

We recently completed a global RNAi screen to identify host factors that are subverted by *S. Typhimurium* to promote its intracellular proliferation. In all, we found 252 human genes that promote *S. typhimurium* growth within MCF-7 cells whose transient absence does not affect host cell viability. This study will facilitate the delineation of the molecular mechanisms underlying the ability of *S. enterica* to persist and replicate within human host cells and provides a large, new class of candidate, anti-microbial targets for controlling *S. enterica* infections, and potentially those caused by other human pathogens as well.

## Results

### Human Epithelial Cell Screen to Identify Host Factors Required for Infection by *S. typhimurium*

**Figure 1:**
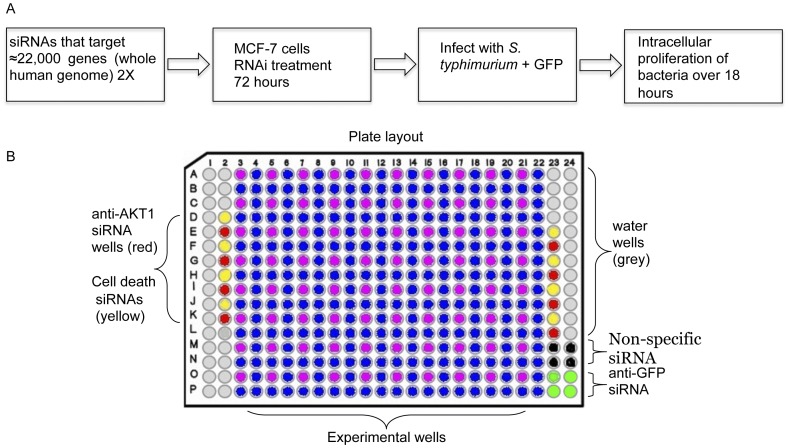
Global RNAi screen of *Salmonella*-infected human epithelial cells. (A) Procedural outline for the screen. (B) The plate layout. Wells that contained siRNAs that result in cell death are in yellow and served as a positive control for transfection. The wells colored red contained anti-AKT1 siRNA, which served as a positive control for the reduction of intracellular *S. typhimurium* growth. The wells colored black contained non-specific siRNA and the wells colored green contained anti-GFP siRNA, which is unrelated to the GFP expressed by the bacteria. Both the non-specific siRNA and anti-GFP siRNA served as negative controls. Water wells, intended to reduce edge effects are in grey, and the experimental wells are in blue and purple.

To identify human genes that modulate the intracellular growth of *S*. *typhimurium,* we developed a GFP fluorescence plate reader-based high throughput RNAi assay with human cells (Fig. 1). We tried intensively to develop a high throughput assay with macrophages but were unsuccessful for a variety of technical reasons, including but not limited to their low transfection efficiency. Ultimately, we resorted to MCF-7 cells, a human epithelial cell line, which can be transfected with >95% efficiency in a high throughput format. For the genome-wide screen, ≈22, 000 predicted genes were targeted with siRNAs arrayed in 384 well plates using a reverse transfection protocol with MCF-7 cells. The screen was performed in a 2×2 format, meaning that each gene was targeted by two independent siRNAs in one well and another two independent siRNAs in a second well on a duplicate plate, reducing the likelihood that off-target effects contributed to the phenotypes. Seventy-two hours following transfection, cells were infected with *S. typhimurium* expressing a plasmid-encoded copy of the GFP at a multiplicity of infection (MOI) of 100 for thirty minutes. The extracellular bacteria were then killed with gentamicin. Eighteen hours later, the number of host cells present in each well and the GFP intensity of infected cells were determined.

Image analysis was carried out by first identifying cell nuclei, which were stained with Hoechst 33342, and extending a boundary a predetermined radial distance from the nuclear border. This region, called a circ, corresponds to an area equivalent to the cell boundary. Only bacteria within this region were analyzed to exclude any residual extracellular bacteria. Targets that modulated intracellular *S. typhimurium* growth were considered hits if they reduced or increased GFP intensity by ≥2.12 standard deviations versus the non-silencing controls on each of the duplicate plates. Thus, the combined Z-score using this two-dimensional analysis was three. We did not compare individual wells to the plate mean to avoid missing legitimate hits as the siRNAs were grouped by functional class. The kinase plate for example would be expected to have numerous hits. Each plate contained several key controls. Some wells contained siRNAs that are lethal to eukaryotic cells. We inspected these wells on all plates to ensure that transfection had occurred. Anti-AKT1 siRNA was also present on every plate and served as a positive control for microbial growth reduction. The plate layout is shown in Figure 1b. The screen strongly enriched the bank of siRNAs for the few that either facilitate or deter *S. typhimurium* intracellular growth in a reproducible fashion in regards to the duplicate plates (Fig. S1).

We ensured that there was no correlation between fluorescence changes and changes in host cell viability by excluding wells that had fewer than 800 cells present. We empirically determined with the first four plates of the screen that there was no longer a correlation between the fluorescence intensity of individual, infected cells and the total number of cells present beyond 800 cells per well (Fig S2). We included gentamicin in our assay, as it is impossible to eliminate the growth of extracellular bacteria without it. We cannot rigorously exclude the possibility that a minority of the genes that appeared to promote microbial growth as measured by a decrease in fluorescence owed their phenotypes to the antibiotic accumulating within cells. However, it is unlikely that very many of them do because in most instances, a cell that had a change in its physiology that resulted in it accumulating gentamicin would be unhealthy.

### Analysis of the Host Network that Facilitates *S. typhimurium* Growth within Human Cells

In all, 252 genes were identified in the primary screen as facilitating intracellular microbial growth, and 39 were identified as normally repressing microbial growth. These genes and their annotations are shown Table S1 and Table S2. The data were further analyzed with IPA (Ingenuity® Systems, www.ingenuity.com). An IPA-generated interaction network including the 252 HSFs is shown in Figure S3. The most central node to the host cell network that promotes *S. typhimurium* growth is the transcription factor NFκB, which among many other things, controls the inflammatory response of host cells. This is perhaps not surprising as a profound inflammatory response in the intestinal epithelium, elicited by the SPI-1 encoded TTSS is a critical feature of *S. typhimurium* pathogenesis [5], [17]. A host response by-product of the inflammation is tetrathionate, a new respiratory electron acceptor that enables *S. typhimurium* to use respiration to compete with fermenting gut microbes, thereby enhancing its transmission [18].

**Figure 2:**
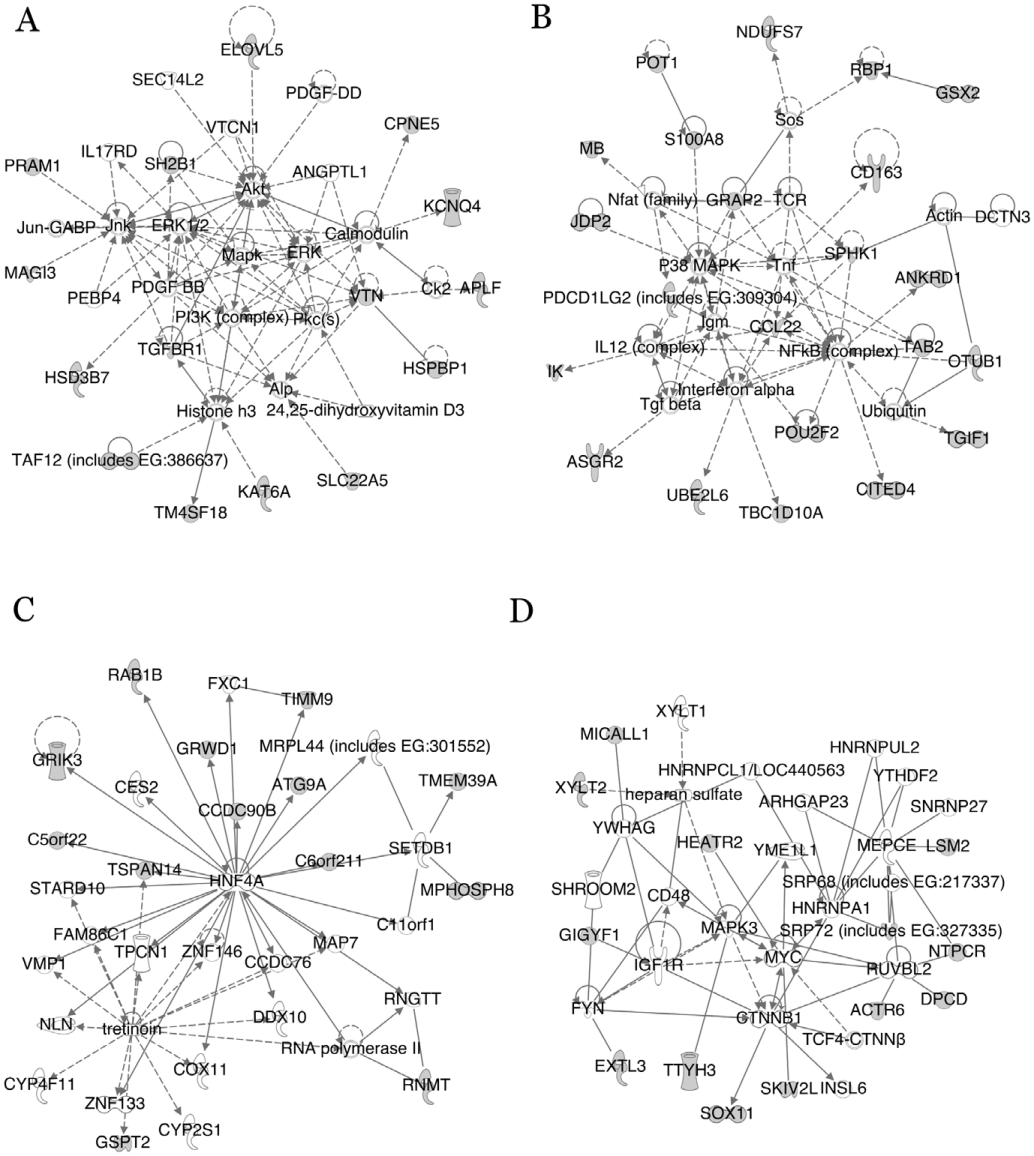
Four prominent sub-networks and associated functions that could plausibly play roles in promoting microbial growth. (A) Cellular development, cellular growth. (B) Cell death. (C) Cell cycle. (D) Carbohydrate metabolism. The shaded molecules are the ones identified in the screen. The others were added by IPA to generate the sub-networks.

**Figure 3:**
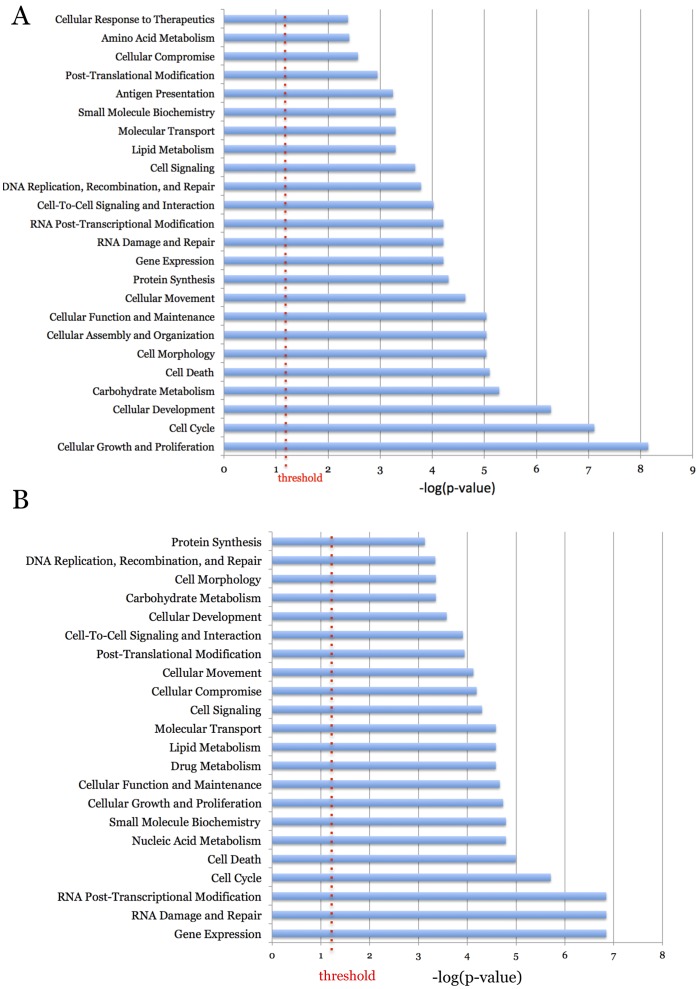
The over-represented molecular and cellular function categories of the entire up and down networks. The entire IPA-generated networks that modulate the intracellular growth of *S. typhimurium* were analyzed for over-represented functional categories. (A) The network that promotes growth. (B). The network that restricts growth. The significance threshold (p = 0.05) is the red, dashed line. The X axis is the –log of the p-values.

In addition to NFκB, centrally located nodes in the host network that permit *S. typhimurium* growth are a series of kinases including MapK, AKT1, p38 MAPK and the pI3K complex. The SPI-1 TTSS is known to lead to the activation of the mitogen-activated protein kinases, ERK, JNK and p38 [5], which are also near the center of the network. These pathways induce signaling to the nucleus and cytokine production. Central to the network also is AKT1, which was previously shown to play a large role in mediating intracellular survival of *S. typhimurium* and *S. typhi* [16]. Four sub-networks that could plausibly play roles in promoting *S. typhimurium* growth within epithelial cells are displayed in Figure 2, including ones that function in cellular growth and development, cell death, cell cycle, and carbohydrate metabolism. Many of the molecules at the center of the global network as well as the molecules in the center of the sub-networks were not identified in the screen. It is likely that depleting the cells of these proteins adversely affected their viability. The IPA-generated, overrepresented molecular function categories for the down network are displayed in Figure 3a, with cellular growth (p-value  = 7×10^−9^), cell cycle (p-value  = 8×10^−8^), cellular development (p-value  = 5×10^−7^) and carbohydrate metabolism (p-value  = 5×10^−6^) being the most prominent. The genes that the various categories are composed of are shown in Table S3. The over-represented molecular function categories from the up network are shown in Figure 3b. Members of the over-represented categories from the up network are listed in Table S4. The categories of cell cycle and cell death were both highly enriched for in both the up and down networks. Also noteworthy, was the identification of numerous components of the proteasome as very strong up hits. Perhaps, depleting cells of proteasome components allows for type III effectors to linger, thereby facilitating enhanced microbial growth.

### The Identification of new HSFs that could Play Plausible Roles in Promoting *S. typhimurium* Growth within Human Cells

**Figure 4:**
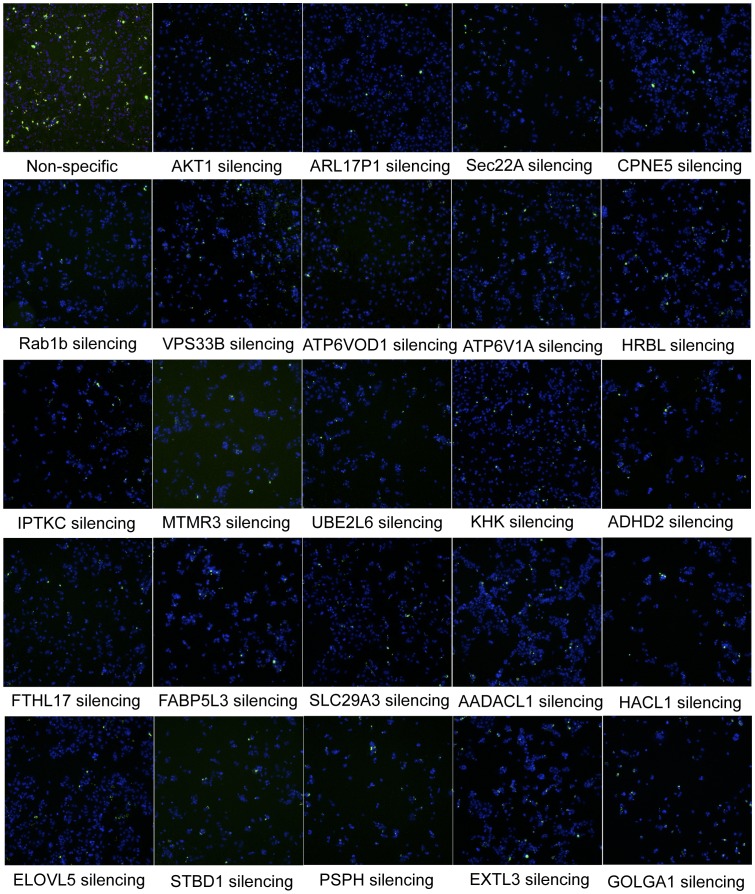
Raw microscopy data from the screen with some of the more interesting hits. Host cells are in blue, *S. typhimurium* is green. Only one representative non-silencing control (upper left corner) and one positive control (anti-AKT1), which is adjacent to the non-silencing control are shown due to space constraints. All of the positive and negative controls appeared similar.

Some of the more interesting, new HSFs identified, which could play plausible roles in mediating bacterial pathogenesis are listed in Table 1, and the raw microscopy data for these hits is shown in Figure 4. These twenty-three hits fall into the general categories of vesicular trafficking, vacuole acidification, signal transduction, lipid synthesis and metabolism, ubiquitylation, carbohydrate metabolism and transport.

**Table 1:** Some of the more interesting hits identified and their characteristics.

Gene symbol	Protein and category	Function	Combined p-values of AB & CD wells
	**Trafficking**		
ARL17P1	ADP-ribosylation factor-like 17 pseudogene 1	Arf GTPase, involved in protein trafficking	0.00063
SEC22A	SEC22 vesicle trafficking protein homolog A (S. cerevisiae)	ER-Golgi vesicle transport	<0.000001
CPNE5	copine V	Membrane trafficking	<0.000001
RAB1B	RAB1B, member RAS oncogene family	Vesicular trafficking	<0.00001
VPS33B	Vacuolar protein sorting 33 homolog B (yeast)	Mediates phagosome-lysosome fusion in macrophages	<0.000001
	**Vacuole acidification**		
ATP6VOD1	ATPase, H+ transporting, lysosomal 13 kDa, V1 subunit G1	Acidifies intracellular compartments	<0.000001
ATP6V1A	V-type proton ATPase catalytic subunit A	Acidifies intracellular compartments	<0.000001
	**Signal transduction**		
HRBL	HIV-1 Rev binding protein-like	Arf gap domain	0.00002
ITPKC	inositol 1,4,5-trisphosphate 3-kinase C	Phosphorylates inositol 2,4,5-triphosphate	0.000004
MTMR3	myotubularin related protein 3	Has phosphatase activity towards phosphatidylinositol-3-phosphate and phosphatidylinositol-3,5-bisphosphate	<0.000001
	**Lipid synthesis and metabolism**		
AADACL1	arylacetamide deacetylase-like 1	May be responsible for cholesterol ester hydrolysis in macrophages	0.0002
HACL1	2-hydroxyacyl-CoA lyase 1	Lipid and fatty acid metabolism	<0.000001
ELOVL5	elongation of long chain fatty acids	Fatty acid, lipid synthesis	<0.000001
	**Ubiquitylation**		
UBE2L6	ubiquitin-conjugating enzyme E2L 6	Catalyzes the covalent attachment of ubiquitin to other proteins.	0.0003
	Carbohydrate metabolism		
KHK	ketohexokinase (fructokinase)	Fructose metablolism	0.00006
AMDHD2	amidohydrolase domain containing 2	hydrolase, carbohydrate metabolism	0.00002
	**Transport**		
FTHL17	ferritin, heavy polypeptide-like 17	Iron transport, oxidoreductase activity	0.0001
FABP5L3	fatty acid binding protein 5-like 3	Lipid binding, transporter activity	0.000005
SLC29A3	solute carrier family 29 (nucleoside transporters), member 3	Membrane, endosomes, imports nucleosides	<0.000001
	**Others**		
STBD1	starch binding domain 1	May have the capability to bind to carbohydrates	0.0003
PSPH	phosphoserine phosphatase	Amino acid biosynthesis	0.00002
EXTL3	exostoses-like 3	Glycosyltransferase	0.000003
GOLGA1	golgi autoantigen, golgin subfamily a, 1	Binds rab6a, involved in maintaining Golgi structure	<0.000001

An incomplete list of some of the genes identified that could play plausible roles in facilitating intracellular microbial growth is shown, with the gene symbols in the first column, the protein and category in the next column, followed by function and the last column contains the combined p-values from the duplicate plates.

We identified at least five HSFs that regulate vesicular trafficking events. Although its functions remain to be fully elucidated, the SPI-2 TTSS likely primarily serves to direct vesicular trafficking events in a manner that renders the *Salmonella*-containing vacuole (SCV) more hospitable. This presumably includes avoiding SCV-lysosome fusion and also enlarging the SCV and importing important metabolic molecules such as amino acids and lipids. Rab1b and Sec22a are a Rab GTPase and SNARE respectively. Both function in the early secretory pathway. Rab1b regulates anterograde traffic between the ER and the Golgi apparatus. A network of Rab GTPases controls phagosome maturation in epithelial cells and is modulated by *S. typhimurium* [19]; however, this network was not known to include Rab1b. *Coxiella burnetii* is known however to recruit Rab1b to its replicative vacuoles and it is required for their proper biogenesis [20]. Similarly, *Legionella pneumophila* recruits Rab1b to the vacuole that it resides within (LCV), which is important for promoting trafficking from the ER to the vacuole and bacterial growth [21]. Sec22a has not previously been implicated in microbial pathogenesis; however, Sec22b is recruited to the LCV and its depletion from cells reduces the replication efficiency of the bacteria [21]. The *M. tuberculosis* secreted protein, PtpA, dephosphorylates the *S. typhimurium* HSF, VPS33b, a regulator of membrane fusion. This interaction inhibits phagosome-lysosome fusion [22]. ARL17P1 and CPNE5 also regulate vesicular trafficking events, but were not previously appreciated as playing roles in microbial virulence [23].

We identified two components of the vacuolar ATPase that acidify intracellular compartments. Numerous pathogens use acid as a cue to escape the vacuole they are within and/or activate virulence gene expression [24]. Acidification of the SCV is important for triggering *S*. *typhimurium* virulence gene expression in J774A macrophages, but not in other macrophage cell lines, and not in HeLa, MDCK or Henle epithelial cell lines [25]. MCF-7 cells, to the best of our knowledge, have never been tested. The effect, if any, of vacuole acidification on *S. enterica* virulence during murine or human infections remains to be determined.

Microbial pathogens often interdict host cell signal transduction pathways and manipulate lipid synthesis and metabolism in ways that promote their virulence. MTMR3 is a phosphatase that acts on lipids with a phosphoinositol (PtdIns) headgroup, with activity towards PtdIns-3-phosphate (PtdIns(3)P) and PtdIns-3,5-bisphosphate (PtdINS(3,5)P_2_) [26]. These activities have been implicated in producing enlarged SCVs that are diverted from the endocytic pathway [27]. ITPKC phosphorylates inositol 2, 4, 5-triphosphate to inositol 2, 4, 5, 6-tetraphosphate. This particular lipid has not previously been implicated in promoting microbial growth. HRBL is an ARF GTPase activator that also was not previously appreciated as playing a role in microbial virulence.

We identified at least three more genes involved in lipid synthesis and metabolism. AADACL1 is believed to be involved in cholesterol ester hydrolysis into their component sterols and fatty acids [28]. It has not previously been implicated in microbial pathogenesis; however, *S. typhimurium* encodes an outer membrane esterase, ApeE, which may be important for virulence as it is not present in *Escherichia coli* [29]. Additionally, ApeE expression is induced by phosphate starvation [30], a condition that *S. typhimurium* likely encounters in the SCV as SPI-2 genes can be strongly induced by it [31]. Elovl5 catalyzes the synthesis of monounsaturated and polyunsaturated very long chain fatty acids, which are structural components of sphingolipids [32], [33]. *S. typhimurium* was shown previously to utilize the SPI-2 TTSS to redirect the Golgi to plasma membrane traffic of a sphingolipid to the SCV [34]. *S. typhimurium* encodes a protein with remarkable similarity to human glucosyl ceramidase and thus could potentially use host sphingolipids as an energy source [35]. Hac1L is involved in lipid and fatty acid metabolism, and was not previously thought to play in role in virulence.

Three additional HSFs that were not previously implicated in microbial virulence are UBE2L6, KHK and AMDHD2. UBE2L6 mediates the covalent attachment of ubiquitin to other proteins and this HSF thus falls into the emerging theme of microbial pathogens manipulating ubiquitylation processes to promote virulence. KHK and AMDHD2 are involved in carbohydrate metabolism and could potentially provide a source of energy to *S. typhimurium*.

We identified at least three proteins that play roles in transport. It stands to reason that intra-vacuolar pathogens such as *S. typhimurium* could benefit from manipulating host proteins involved in transport to obtain host factors that facilitate the growth of the microbe within the normally, inhospitable SCV. FTHL17 is involved in iron transport. The iron withholding mechanisms of host cells are essential for controlling microbial infections. Successful pathogens, such as *S. typhimurium* have developed numerous, clever strategies for circumventing this innate host defense. One among numerous bacterial counter-measures is the *sitABCD* iron acquisition operon of *S. typhimurium* that is induced following invasion of the intestinal epithelium [36]. FTHL17 has never been shown before to support microbial growth; however, it is reasonable to think that it could as it is involved in iron transport. FABP5L3 has likewise not been implicated in microbial pathogenesis previously. However, as it transports fatty acids with a high specificity, which could be useful to *S. typhimurium,* it is not unreasonable to think that it does play a role in supporting virulence. SLC29A3 belongs to the SLC29A transporter family. It localizes to enodosmes and lysosomes normally where it mediates the influx and efflux of nucleosides [37], [38]. Rickettsial organisms, which are obligate intracellular parasites, are known to scavenge nucleosides from infected host cells [39]; however, S. *typhimurium* as a free-living organism can synthesize its own nucleosides. But, it is possible that *S. typhimurium* augments endogenous nucleosides with ones from the host cell for DNA and RNA synthesis and additionally, nucleoside tri-phosphates could of course serve as an energy source.

Four additional genes among many identified in this screen that could plausibly play roles in permitting the growth of *S. typhimurium* within host cells are STBD1, PSPH, EXTL3 and GolgA1. STBD1 is interesting in that it has a microbial starch-binding domain, which is involved in glycogen metabolism. Glycogen is a polysaccharide that is the principal storage form of glucose in human cells, and could serve as an energy source for the bacteria residing within the SCVs. STBD1 is in fact normally localized to late endosomes and lysosomes where it anchors glycogen to the membranes [40], [41]. PSPH catalyzes the last step in the biosynthesis of serine from carbohydrates. It seems possible that the bacteria scavenge serine from the host cells, considering the presumably stringent nutrient conditions of the SCV. *S. typhimurium* infection has been shown to up-regulate the expression of the cationic amino acid transporters mCAT1 and mCAT2B, which regulate the availability of arginine within macrophages. Interestingly, during infections they localize in close proximity to the SCV. The intra-vacuolar bacteria then use their own arginine transporter to acquire the amino acid [42]. *S. typhimurium* encodes a putative serine transport protein, SdaC [43]. Perhaps manipulating host amino acid synthesis and localization will prove to be a common strategy deployed by pathogens. EXTL3 is a probable glycosyltransferase involved in the metabolism of glycan and heparin sulfate, which are well known for their roles in mediating pathogen attachment and subsequent invasion of host cells [44]. Glycans have not however previously been implicated in promoting the intracellular growth of microbes. It is possible that EXTL3 creates useful energy sources for *S. typhimurium.* GolGA1 is a member of a family of Golgi-targeted coil-coil proteins that bind Rab6a [45].

### Comparison of the Host Networks that Promote the Intracellular Growth of *S. typhimurium* and *M. tuberculosis*

We compared our results to the only other global RNAi screen performed on human cells infected with bacteria. This screen analyzed THP-1 human macrophage-like cells infected with *M. tuberculosis* [46]. This study identified 272 human HSFs for *M. tuberculosis.* An IPA-generated network of these HSFs yielded 501 molecules (data not shown) whereas the *S. typhimurium* network is composed of 474 molecules. Interestingly, they have 49 molecules in common. The union of the two networks is show in Figure S3, and the members are listed in Table S5. The most over-represented molecular function category for the 49 molecules that the two networks share is autophagy (p-value <4.7×10^−11^).

## Discussion

Although much is known about the microbial virulence factors that *S. enterica* deploys to invade and subsequently grow within host cells, relatively little is known about the cellular processes that modulate virulence. This genome-wide screen identifies the human genes that are exploited by *S. enterica* to facilitate intracellular growth. In addition to providing a wealth of new information that will ultimately facilitate the unraveling of many of the molecular mechanisms underlying the ability of *S. enterica* to persist and proliferate within human cells, all of the 252 factors identified in this study are candidates for a new class of anti-microbial targets.

The host gene network derived from this study that facilitates the intracellular growth of *S. typhimurium* within human epithelial cells has significant overlap with the one that promotes the growth of *M. tuberculosis* within human macrophages. This suggests a conservation of basic processes between the two cell types and may point to new drug targets that could have broad-spectrum activity. It is interesting that the shared molecules, for the most part, are the central nodes of both networks, perhaps revealing some of the shared, core, underlying logic through which different pathogens manipulate host cells. The union of the two networks contains at least 18 genes that regulate autophagy. Curtailing the growth of intracellular pathogens with autophagy is an ongoing theme in host-pathogen interplay. Following internalization, *S. typhimurium* can damage the SCV with its SPI-1 encoded TTSS. This damage is believed to produce an intracellular bacterial population that is targeted by the autophagy system of the host cell [47]. In fact, cells deficient in autophagy permit more intracellular *S*. *typhimurium* growth than normal cells, due to the creation of a population of bacteria in the cytosol that can replicate rapidly in this nutrient-rich environment [47]. Kumar *et*. *al* reported that the most conserved members of the host survival networks that permit the persistence of diverse strains of *M*. *tuberculosis* function through the regulation of autophagy [46]. Group A *Streptococcus pyogenes* deploys Streptolysin O to lyse phagosomes and gain access to the cytosol, following which autophagy limits its growth [48]. *Rickettsiae* spp. also gain access to the cytosol and can be targeted by autophagy for destruction [49], [50]. Thus, it is possible that negative regulators of autophagy could serve as anti-microbial targets for numerous pathogens. However, as some of the 18 genes that the *S. typhimurium* and *M*. *tuberculosis* host survival networks share that regulate autophagy can promote it, and as these genes have numerous, and in some instances diverse functions within host cells, further work will be required to clarify the role of these genes in permitting intracellular, microbial growth.

The most over-represented molecular function categories from the down network, namely cell growth and development, cell cycle, and carbohydrate metabolism are consistent with some of the general themes of host-microbe interactions. It was interesting that the categories of cell death and cell cycle were significantly over-represented in both the up and down hits. Microbial pathogens are well known for their abilities to kill cells by triggering apoptotic pathways as well as causing necrotic death and conversely to block apoptosis. Pathogens trigger cell death in order to avoid killing by professional phagocytes, or to escape a cell that cannot support additional microbial growth, or to modulate the immune response. Pathogens can also activate cell survival pathways and interdict apoptotic signaling in order to create a safe, replicative niche [51]–[54]. In the screen described here, it is likely that the genes identified in the up hits promoted cell death whereas the ones in the down hits suppressed it. In addition to apoptotic pathway manipulation, numerous pathogens are known to manipulate the host cell cycle machinery in various ways, sometimes blocking the cell cycle at transition phases and also sometimes forcing progression through the cell cycle [55]. In future work, it will be interesting to learn more about why so many of the up and down hits influence cell cycle and to dissect how *S. enterica* manipulates these genes to its advantage.

Several HSFs identified warrant further consideration. The hits in the trafficking category may be of further interest as understanding exactly how they subvert the endocytic and exocytic pathways would greatly enhance our understanding of SCV biogenesis, which is intimately tied to virulence. We likely did not identify more host proteins known to play roles in regulating intracellular trafficking as it was observed that the depletion of single ER to Golgi transport proteins rarely decreased *L. pneoumophila* intra-vacuole replication. Combination knock-downs on the other hand did reduce *L. pneoumophila* growth, suggesting that membrane traffic funnels into the vacuole from multiple sources [23].

MTMR3 is an intriguing HSF that defies easy classification as it is involved in lipid metabolism, signal transduction and also regulating the endocytic pathway. *Saccharomyces cerevisiae* cells deficient in PtdIns(3,5)P_2_ synthesis exhibit grossly enlarged vacuoles analogous to the spacious phagosomes *S. typhimurium* resides within [56]. The levels of PtdIns(3)P and PtdIns(3,5)P_2_ are established by the activities of the PtdIns(3)P 5-kinase, Fab1, and the PtdIns(3,5)P_2_ phosphatase, FIG4, and also by members of the myotubularin lipid phosphatase family. It was previously proposed that the PtdIns(3,5)P_2_ phsophatase activity of the *Salmonella* SPI-1 type III effector SopB may play a role analogous to FIG4, preventing the transition of PtdIns(3)P to PtdINS(3,5)P2, thereby resulting in enlarged vesicles and diverting the SCV from the endocytic pathway [27]. Depleting cells of MTMR3 would remove one of the mechanisms through which PtdINS(3,5)P_2_ is hydrolyzed as well as removing PtdIns(3)P substrates for Fab1. On the other hand, high levels of of PtdIns(3)P in the membrane of SCVs was proposed to stimulate homotypic vesicle fusion with other PtdIns(3)P containing empty vesicles formed during bacterial infection to promote virulence, attributable to the PtdIns(3,4,5)P_3_ phosphatase activity of SopB. SopB and MTMR3 may have somewhat antagonistic activities, which allow for the optimal manipulation of vesicular fate. Clearly, more work will be required to fully understand how MTMR3 is subverted by *S. typhimurium* to promote its growth within human cells.

It is perhaps not surprising that we identified a ubiquitin conjugating enzyme as an HSF considering that *S. typhimurium* secretes multiple SPI-1 and SPI-2 effectors into infected cells that interfere with ubiquitin-related processes. SopA and SspH2 are E3 ligases, whereas SseL is a deubiquitinase required for macrophage killing through the delayed pathway [52], [53]. The effectors SopA, SopE, SptP and SopB are all ubiquitinated in ways that provide spatiotemporal regulation and diversification of function [57], [58]. Clearly, manipulating ubiquitin with secreted bacterial proteins and with host proteins is a major feature of *S. typhimurium* pathogenesis. It will be interesting to determine in future work how UBE2L6 fits into the bigger picture.

GolgA1 is another interesting HSF in that it could conceivably be involved in tethering the SCV to the Golgi apparatus, a phenomenon that is critical to *S. typhimurium* virulence [59]. In infected epithelial cells, SCVs migrate to a perinuclear location where they become surrounded by membranes of the Golgi network. This subcellular localization requires the SPI-2 effector SseG. Both the N- can C-terminal domains of SseG are exposed on the cytosolic face of the SCV. *sseG* mutants are unable to localize the vacuoles they reside within to the Golgi and are unable to multiply. The Golgi-targeting sequence of SseG appears to be novel as there is no similarity between SseG and either prokaryotic or eukaryotic Golgi targeting sequences [59]. The family of proteins that GolgA1 belongs to was proposed to function in Rab6-regulated membrane tethering events [45]. Although purely speculative, it is interesting to consider that SseG might interact with GolgA1, tethering the SCV to the Golgi apparatus. Alternatively, GolgA1 could play a role in manipulating Golgi structure or perhaps exocytic events in a way that benefit the bacteria within the SCV.

With a genome-wide RNAi-based forward genetic screen, we have identified 291 host factors that either promote or repress the growth of *S. typhimurium* within human epithelial cells. Additionally, this study identifies 49 molecules that are needed by both *S. typhimurium* and *M. tuberculosis* to grow optimally within human cells. In future work, it will be important to determine what the core group of HSFs is that are required for both *S typhimurium* and *S. typhi* survival within both epithelial cells and macrophages, and further which ones are required by multiple intracellular pathogens. This may help us understand the shared logic though which all pathogens manipulate host cells, and allow for the rational design of new drugs for treating infectious disease. It will also be interesting to study some of the HSFs identified in detail. The genome-wide results from our human RNAi screen provide us with some new insights into the *Salmonella* intracellular lifestyle and serve as an important starting point for delineating the molecular mechanisms through which intracellular pathogens manipulate host cells.

## Materials and Methods

### Bacterial and Eukaryotic Cell Growth Conditions

*S. typhimurium* 14028s carrying a derivative of pACYC184 which expresses the GFP was grown in Luria Bertani broth (LB; Sigma-Aldrich, St. Louis, MO) for 18 hours at 37°C with agitation. It was then diluted 1∶33 in fresh LB and subcultured for 3.5 hours at 37°C with agitation to induce SPI-1 expression prior to infection of MCF-7 cells (ATCC, Manassas, VA). MCF-7 cells were grown in Iscove’s Modified Dulbecco’s Medium (IMDM; Invitrogen, Carlsbad, CA) supplemented with 7.5% fetal bovine serum (FBS; Sigma-Aldrich) without antibiotics. MCF-7 cells were maintained in a humidified tissue culture incubator at 37°C in 5% CO_2_ and subcultured every four days at 8×10^4^ cells/cm^2^.

### Infections and Gentamicin Protection Assay

MCF-7 cells were infected with *S. typhimurium* at an MOI of 100, following which cells were centrifuged at 230 relative centrifugal force for 10 minutes at room temperature. Infected cells were incubated at 37°C in 5% CO_2_ for 30 minutes to allow for invasion and then washed two times. After washing, the cells were incubated in IMDM supplemented with gentamicin (Gibco, Carlsbad, CA) at a concentration of 100 µg/mL for one hour at 37°C in 5% CO_2_ to prevent further invasion and to eliminate extracellular bacteria. Following the gentamicin kill, cells were washed two times and incubated in IMDM supplemented with gentamicin at a concentration of 10 µg/mL and incubated at 37°C in 5% CO_2_ for 18 hours before preparing plates for imaging.

### High-throughput siRNA Screen of Human Genome

A siRNA library was obtained from the Duke University RNAi screening facility targeting the human genome (Human whole-genome siRNA library v1.0; Qiagen, Valencia, CA) with four individual siRNAs targeting each gene arrayed in two sets, AB and CD, in a 2×2, parallel plate format. MCF-7 cells were seeded at a density of 3000 cells per well in 384 well clear flat bottom plates (Corning 3712; Corning, Lowell, MA). Reverse transfections were performed using 0.05 µL Dharmafect 4 (Dharmacon, Lafayette, CO) and 20 nM siRNA perwell in a total volume of 50 µL. Transfection efficiency was optimized utilizing AllStars Hs Cell Death Control siRNA (Qiagen) and was >95% under these conditions. Seventy-tw0 hours after transfection the cells were infected with *S. typhimurium* expressing the GFP that were induced for SPI-1 expression. Infected MCF-7 cells were fixed eighteen hours after infection with paraformaldehyde (Sigma-Aldrich) in 1X PBS (Invitrogen) for 1 hour. Nuclei were stained with Hoechst 33342 (Sigma-Aldrich) and wells were imaged and analyzed with the Cellomics ArrayScan VTI High Content imaging system (Thermo Scientific, Pittsburgh, PA). Only infected cells were analyzed to exclude any residual extracellular bacteria. The average intensity of all circs within a well was measured. These were then compared with the mean circ spot average intensity of the negative controls. We compared the experimental wells to the wells containing non-specific siRNA, which proved to be a more conservative approach than comparing them to the anti-GFP wells. The anti-GFP siRNA was unrelated to the GFP expressed by the bacteria.

### Bioinformatics

The gene sets identified in our screen were manually curated with Uniprot (http://www.uniprot.org/). Other bioinformatics analyses were performed with IPA (Ingenuity® Systems, www.ingenuity.com). A data set containing gene identifiers was uploaded into the application. Molecules with IDs that could be mapped were overlaid onto a global molecular network developed from information contained in the Ingenuity Knowledge Base. Networks of network eligible molecules were then algorithmically generated based on their connectivity, and merged. Functional analysis identified the molecular and cellular functions that were most significant to the entire network that was generated. Right-tailed Fisher’s exact test was used to calculate a p-value determining the probability that each molecular function assigned was due to chance alone. The network generated is a graphical representation of the molecular relationships between molecules. Molecules are represented as nodes, and the biological relationship between two nodes is represented as an edge (line). All edges are supported by at least one reference from the literature, from a textbook, or from canonical information stored in the Ingenuity Knowledge Base.

## Supporting Information

Figure S1 **Distribution of the standard deviations of the average infected cell fluorescence intensity from each well in the screen.** (A) The standard deviations of all experimental wells from the mean of the non-specific siRNA negative controls are plotted. The cutoffs for accepting hits were plus 3 or minus 3, which are indicated with arrows. Thus, there was a strong enrichment in the screen. (B) The standard deviations of the hits are plotted. The means for the negative controls should be around 0, 0.
(TIF)

Figure S2 **Changes in fluorescence were not attributable to changes in host cell viability.** (A) We observed a linear relationship between average cell fluorescence intensity and cell numbers up to 800 cells/well. (B) Above 800 cells/well there was no longer a linear relationship between cell numbers and the average fluorescence intensity of infected cells. The lack of linearity above 800 cells/well reveals that increasing the viable cells beyond this point does not increase microbial growth in individual, infected cells, decoupling host cell viability from microbial growth.
(TIF)

Figure S3 **The network generated from the 252 HSFs identified in this study.** Molecules are represented as nodes and lines represent interactions between them. The molecule labels in blue are the union of the *S. typhimurium* and *M. tuberculosis* networks.
(TIF)

Table S1 **The uniprot derived gene annotations and the raw numerical data for the down hits.**
(XLSX)

Table S2 **The uniprot derived gene annotations and the raw numerical data for the up hits.**
(XLSX)

Table S3 **The members of the over-represented molecular function categories for the down hits are listed.**
(XLSX)

Table S4 **The members of the over-represented molecular function categories for the up hits are listed.**
(XLSX)

Table S5 **The molecules that the union of the** ***S*** **.** ***typhimurium*** **and** ***M*** **.** ***tuberculosis*** **host survival networks are composed of are listed.**
(XLSX)
