# Supplementary figures and images for: Human Genome-Wide RNAi Screen for Host Factors That Modulate Intracellular Salmonella Growth
Source: PLoS One. 2012 Jun 11;7(6):e38097. doi: 10.1371/journal.pone.0038097 (PMC3372477; doi:10.1371/journal.pone.0038097)

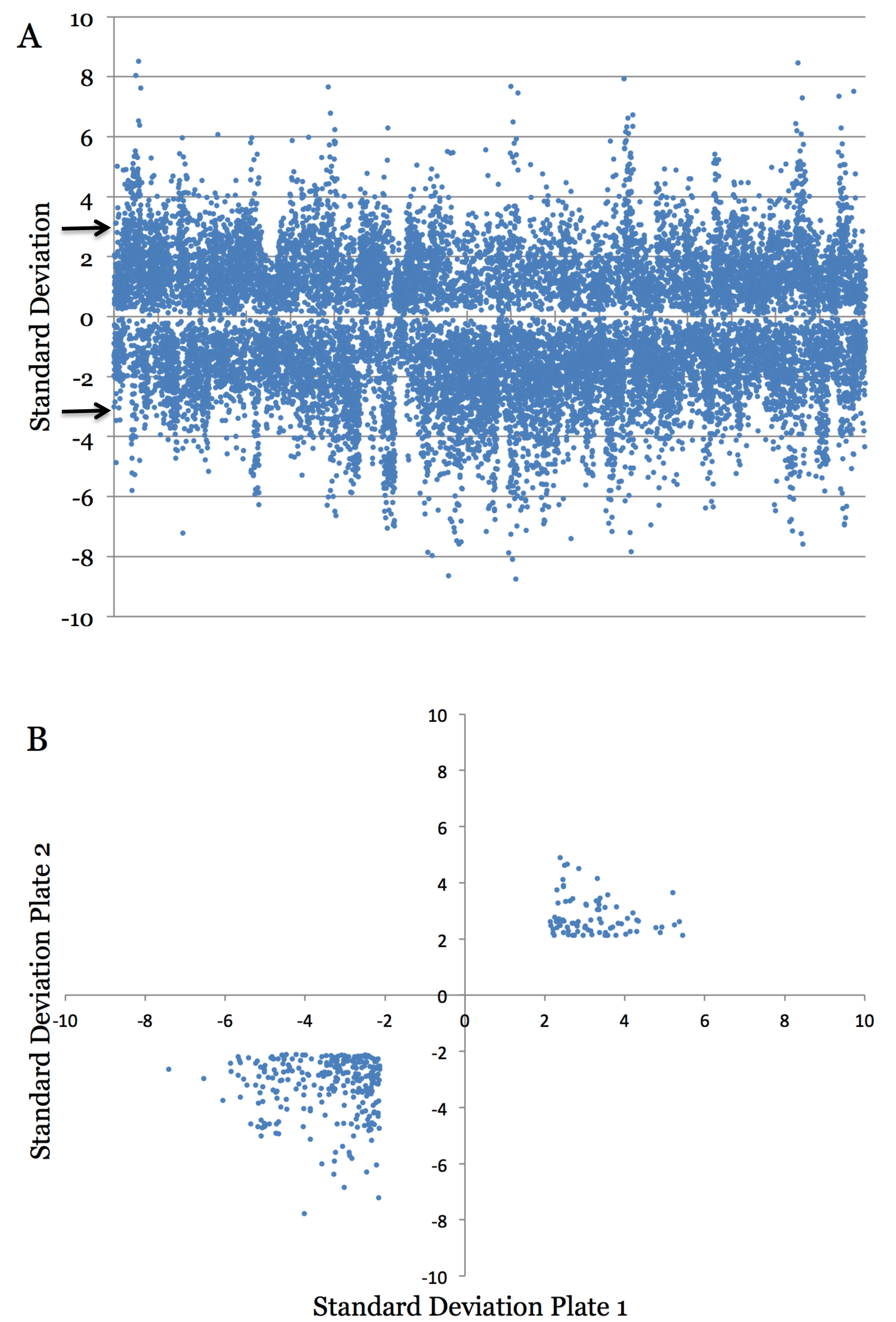

Supplement: Figure S1 — Distribution of the standard deviations of the average infected cell fluorescence intensity from each well in the screen. (A) The standard deviations of all experimental wells from the mean of the non-specific siRNA negative controls are plotted. The cutoffs for accepting hits were plus 3 or minus 3, which are indicated with arrows. Thus, there was a strong enrichment in the screen. (B) The standard deviations of the hits are plotted. The means for the negative controls should be around 0, 0. (TIF) [file pone.0038097.s001.tif]

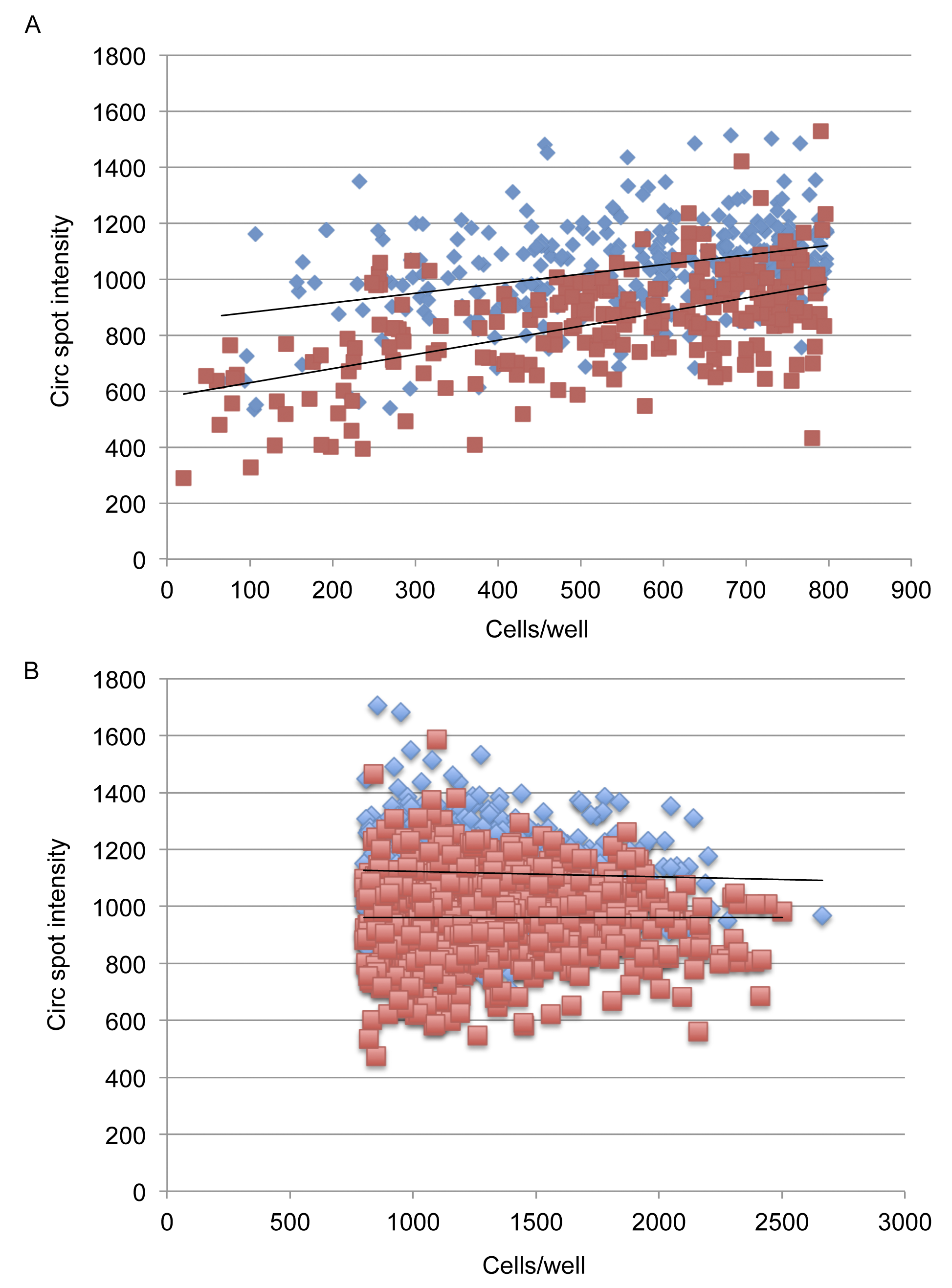

Supplement: Figure S2 — Changes in fluorescence were not attributable to changes in host cell viability. (A) We observed a linear relationship between average cell fluorescence intensity and cell numbers up to 800 cells/well. (B) Above 800 cells/well there was no longer a linear relationship between cell numbers and the average fluorescence intensity of infected cells. The lack of linearity above 800 cells/well reveals that increasing the viable cells beyond this point does not increase microbial growth in individual, infected cells, decoupling host cell viability from microbial growth. (TIF) [file pone.0038097.s002.tif]

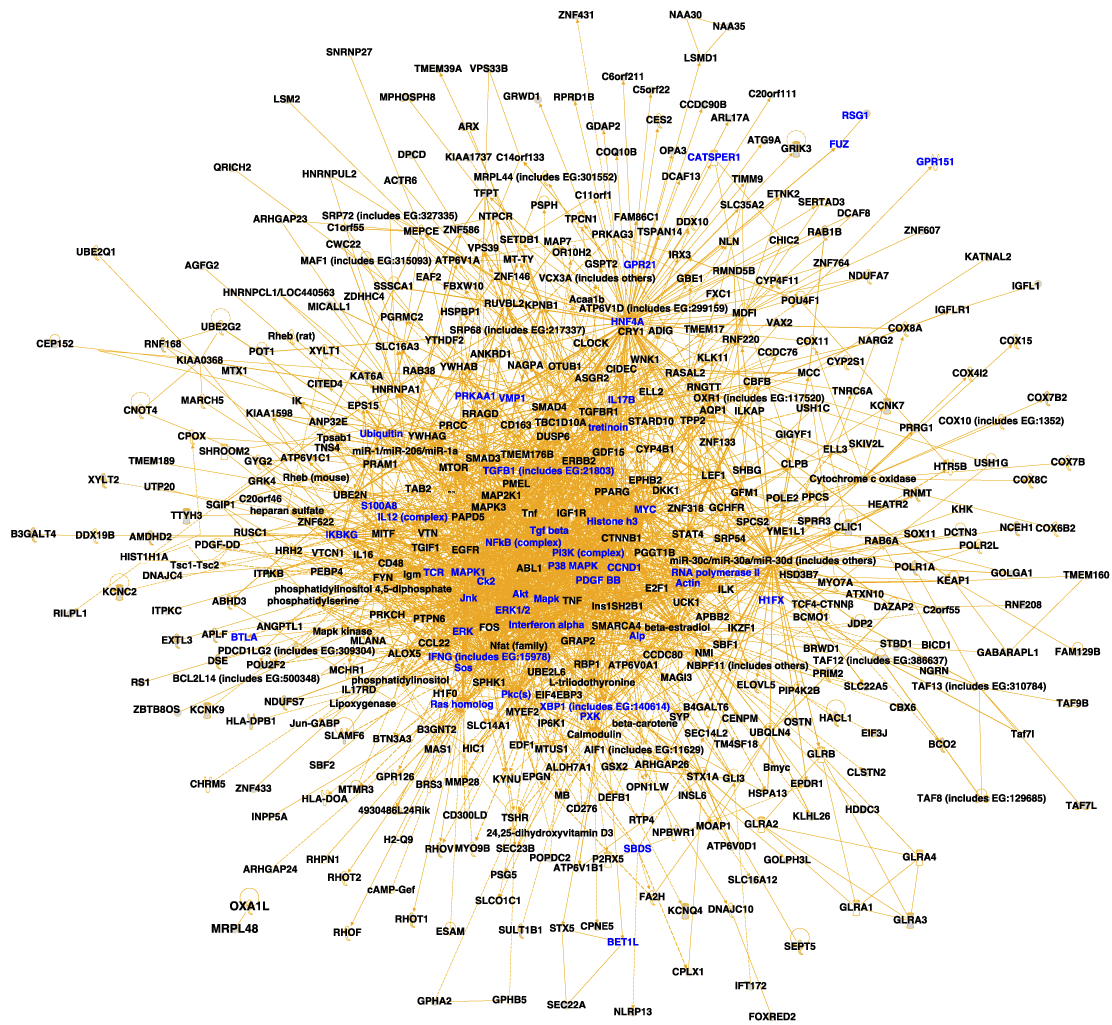

Supplement: Figure S3 — The network generated from the 252 HSFs identified in this study. Molecules are represented as nodes and lines represent interactions between them. The molecule labels in blue are the union of the S. typhimurium and M. tuberculosis networks. (TIF) [file pone.0038097.s003.tif]
